# Collagenous Fibroma (Desmoplastic Fibroblastoma) with Vertebral Body Erosion

**DOI:** 10.1155/2009/682687

**Published:** 2009-05-27

**Authors:** Vladimir Osipov, Guillermo F. Carrera

**Affiliations:** ^1^Department of Patholog, Medical College of Wisconsin, 9200 West Wisconsin Avenue, Milwaukee, 53226, USA; ^2^Department of Radiology, Medical College of Wisconsin, 9200 West Wisconsin Avenue, Milwaukee, 53226, USA

## Abstract

Collagenous fibroma (desmoplastic fibroblastoma) is a recently described rare benign tumor affecting mainly males in the fifth through seventh decades. This tumor occurs predominantly in the peripheral sites, with predilection for upper and lower extremities. The patients present with a painless mass of involving subcutis, with one quarter of all cases involving skeletal muscle. Both radiographically and histologically these tumors are well-circumscribed small lesions from one to several centimeters in diameter, though lesions as large as 20 cm have been reported. We report a case of collagenous fibroma presenting symptomatically as a 2.4 cm mass within the pedicle and adjacent transverse process of the L5 vertebral segment. Bone erosion by desmoplastic fibroblastoma is very rare event. This tumor should be in the differential diagnosis of the soft tissue lesions presenting with bony erosion.

## 1. Introduction

Collagenous fibroma (desmoplastic fibroblastoma) is a recently defined entity first described by Evans in 1995 [1]. Since then, fewer than 100 cases have been reported with the largest series published by Miettinen and Fetsch [2]. Collagenous fibroma is a benign fibrous soft tissue tumor typically arising in the subcutaneous tissue or skeletal muscle of adults. A wide age range is affected, but it is most common in the fifth through seventh decades. Men are affected four times more commonly than women. Collagenous fibroma has a wide anatomic distribution affecting mainly extremities and presents as a slowly growing, painless mass, ranging from 1 to 20 cm in maximum dimension [2]. Surgery is the treatment of choice, with no reported tumor recurrences. Differential diagnosis includes desmoid tumor, Gardner fibroma, and nuchal-type fibroma. Cases with cytogenetic abnormalities have been reported suggesting a neoplastic nature for this tumor [3]. Bone erosion is an extremely rare event with only one case previously published [4].

## 2. Case Report

**Figure 1:**
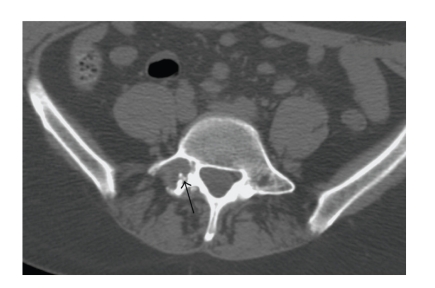
CT image shows a well-circumscribed, minimally expansile lytic lesion in the right pedicle and adjacent transverse process of L5. Tiny densities (arrow) represented bony fragments from adjacent degenerated facet joint.

**Figure 2:**
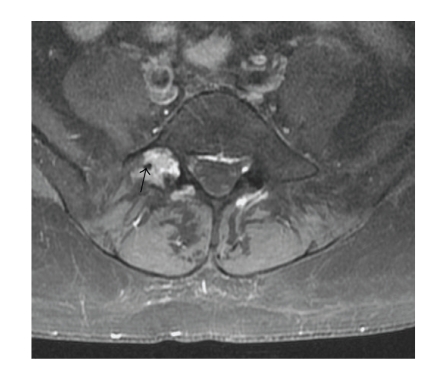
Fat-saturated T1 post-Gadolinium images show mixed intermediate signal in the lesion with moderate heterogeneous generalized enhancement. There is no adjacent edema or reaction in the bone or soft tissues. Small areas of low signal (arrows) are present.

**Figure 3:**
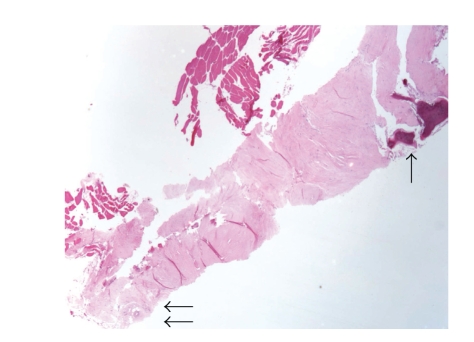
Full length Tru-cut biopsy of the tumor. Note the entrapment of the skeletal muscle and adipose tissue at the periphery (double arrow) and involvement of the bone at the other end (single arrow). (H&E, 40X).

**Figure 4:**
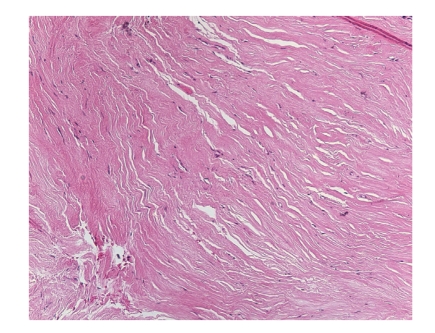
Hypocellular densely collagenous stroma of the tumor. (H&E, 400X).

The patient, a 56-year-old woman, presented with right L5 radicular pain, worse at night, which subsequently improved with Neurontin (Gabapentin) therapy. Imaging evaluation including plain films, CT, and MRI showed a lytic, minimally expansile intraosseous soft-tissue mass occupying the right L5 pedicle and extending into the adjacent transverse process (Figure 1). The lesion measured 2.4 cm in greatest dimension. The lesion was lytic, expansile and well-defined on CT. MRI showed heterogeneous intermediate signal intensity on T2 and T1 weighted images with scattered areas of low signal intensity on both sequences, and diffuse minimally heterogeneous enhancement following intravenous Gadolinium (Figure 2). There was degenerative change in the adjacent facet joint. Differential diagnosis included osteoblastoma, giant cell tumor, and metastasis. A CT-directed core biopsy was performed using a 14 gauge coaxial system. Histology revealed a uniformly paucicellular tumor (Figure 3). There were widely spaced, bland spindle to stellate shaped cells embedded in a dense fibrous stroma with homogeneous collagen bundles (Figure 4). There was no cytoatypia. Mitotic figures and necrosis were absent. Focally, fragments of unremarkable bone were present. The tumor showed entrapment of the adipose tissue and skeletal muscle at the periphery. Vimentin immunohistochemical stain showed positivity within the spindle cells. Smooth muscle actin showed very faint focal positivity. Congo red histochemical stain was negative, excluding the possibility of amyloidosis. Two years later, patient had no evidence of recurrence and was symptoms-free.

## 3. Discussion

 The most important information related to the lesions in the differential diagnosis of collagenous fibroma is summarized in Table 1, created using the information found in the recent issue of WHO Tumors of Soft Tissue and Bone [5].

 One additional differential diagnosis not included in this table is a “burned-out” phase of nodular fasciitis. Whether one wishes to interpret desmoplastic fibroblastoma as a “burnt-out” phase of a nodular fasciitis is probably irrelevant for all practical purposes. However, the nodular fasciitis can present similarly to the collagenous fibroma. One of the most recent papers [6] describes a case of a superficial cortical erosion of a long bone.

Our report is unique for several reasons. Our patient presented with pain, while the vast majority of collagenous fibromas are asymptomatic. In addition, bone erosion is a very rare event with only one case previously described [4]. The imaging findings of this lesion match those reported in soft tissue collagenous fibroma [7, 8]. The plain film findings are nonspecific, and in our case, the lesion consisted of a lytic, minimally expansile focus suggesting slow, nonaggressive growth. No calcifications were visible on radiography or CT. The MRI findings included heterogeneous intermediate signal on both fat and fluid sensitive sequences, minimally heterogeneous diffuse enhancement and focal (in our lesion, very small) areas of persistently low signal suggesting regions of dense acellular collagen aggregates. 

Since a significant component of the tumor was at least contiguous with the adjacent soft tissue, the intraosseous component in our case probably represents an erosion of the vertebral body by the tumor. Interestingly, a peripheral entrapment of the skeletal muscle and adipose tissue was noted histologically (Figure 3), a feature present in over one half of the tumors reported by Miettinen and Fetsch [2]. Other authors also noted this feature [9]. In one report a collagenous fibroma of alveolar bone was reported [10], but the authors stated that there was no osseous involvement with only osteogenic reaction of the subjacent bone. 

In summary, collagenous fibroma should be in the differential diagnosis of a well-circumscribed lesion with intraosseous component. Simple excision is recommended in symptomatic patients.

## Figures and Tables

**Table 1:** Summary of the clinical, radiographic, and pathological characteristics of the tumors in the differential for collagenous fibroma.

Tumor type	Age, gender	Location	Imaging	Histology
Collagenous fibroma	Mainly males, between fifth and seventh decades of life	Extremities	Well-circumscribed	Hypocellular, with prominent collagenous background, entrapment of the soft tissue at the periphery.
Desmoid tumor (extra-abdominal)	Puberty-middle age, no gender predilection	Shoulder, chest wall, back, thigh, head and neck	Poorly defined	Variably cellular stroma containing compressed or distorted blood vessels
Gardner's fibroma	Children, adolescents, no gender predilection	Paraspinal region, back, chest wall, flank, head and neck, extremities	Poorly defined	Hypocellular, entrapment of the soft tissue at the periphery. Not vascular.
Nuchal-type fibroma	Third through fifth decades of life, male predilection	Classically in the posterior neck	Poorly defined	Hypocellular, with haphazardly arranged collagen fibers, nerve twigs.
